# Role of *LOC_Os01g68450*, Containing DUF2358, in Salt Tolerance Is Mediated via Adaptation of Absorbed Light Energy Dissipation

**DOI:** 10.3390/plants11091233

**Published:** 2022-05-02

**Authors:** Chutarat Punchkhon, Panita Chutimanukul, Ratchata Chokwiwatkul, Triono Bagus Saputro, Aleel K. Grennan, Nuria De Diego, Lukáš Spíchal, Supachitra Chadchawan

**Affiliations:** 1 Program in Biotechnology, Faculty of Science, Chulalongkorn University, Bangkok 10330, Thailand; chutarat_tom@hotmail.com (C.P.); trionobsaputro@gmail.com (T.B.S.); 2 Center of Excellence in Environment and Plant Physiology, Department of Botany, Faculty of Science, Chulalongkorn University, Bangkok 10330, Thailand; panita.chu@biotec.or.th (P.C.); ratchata.chok@gmail.com (R.C.); 3 National Center for Genetic Engineering and Biotechnology, National Science and Technology Development Agency, 113 Phahonyothin Rd. Khlong Nueng, Khlong Luang, Pathumthani 12120, Thailand; 4 Program in Biological Science, Faculty of Science, Chulalongkorn University, Bangkok 10330, Thailand; 5 Biology Department, Worcester State University, Worcester, MA 01602, USA; agrennan@worcester.edu; 6 Centre of Region Haná for Biotechnological and Agricultural Research, Czech Advanced Technology and Research Institute, Palacký University Olomouc, Šlechtitelů 27, CZ-783 71 Olomouc, Czech Republic; nuria.de@upol.cz (N.D.D.); lukas.spichal@upol.cz (L.S.); 7 Omics Science and Bioinformatics Center, Faculty of Science, Chulalongkorn University, Bangkok 10330, Thailand

**Keywords:** rice, *at1g65230* mutant line, light-harvesting complex, photosynthetic pigment, salt stress, phiPSII, electron transport rate, stomatal conductance

## Abstract

Salt stress affects plant growth and productivity. In this study we determined the roles of eight genes involved in photosynthesis, using gene co-expression network analysis, under salt-stress conditions using Arabidopsis knockout mutants. The green area of the leaves was minimum in the *at1g65230* mutant line. Rice *LOC_Os01g68450*, a homolog of *at1g65230*, was ectopically expressed in the *at1g65230* mutant line to generate revertant lines. Under salt stress, the revertant lines exhibited significantly higher net photosynthesis rates than the *at1g65230* mutant line. Moreover, the operating efficiency of photosystem II (PSII) and electron transport rate of the revertant lines were higher than those of the wild type and *at1g65230* mutant line after 10 days of exposure to salt stress. After this period, the protein PsbD–the component of PSII–decreased in all lines tested without significant difference among them. However, the chlorophyll *a* and *b*, carotenoid, and anthocyanin contents of revertant lines were higher than those of the mutant line. Furthermore, lower maximum chlorophyll fluorescence was detected in the revertant lines. This suggests that *LOC_Os01g68450* expression contributed to the salt tolerance phenotype by modifying the energy dissipation process and led to the ability to maintain photosynthesis under salt stress conditions.

## 1. Introduction

Salt stress is one of the most common abiotic stresses that affect plant growth and productivity. In 2019, approximately 1.125 MH of land was highly saline, and that area is likely to increase by 1–2% each year [1]. Salt stress affects plants via osmotic and oxidative stress, inhibition of nutrient absorption, ion toxicity, and metabolism imbalance. These affect biological processes including growth, photosynthesis, protein synthesis, and protein and phospholipid metabolism. Under salt-stress conditions, plants close their stomata to prevent water loss from leaves, resulting in decreased carbon assimilation. Additionally, salt stress induces the production of reactive oxygen species (ROS) that results in thylakoid disruption and chlorophyll degradation. These salt-stress-induced plant responses suppress photosynthesis [2,3], thereby reducing crop growth and yield.

Salt and drought stresses, which both induce water deficit in plants, are abiotic. These stresses have similar effects on plants during the early stages of stress. Prolonged salt stress induces hyperionic and hyperosmotic stresses in plants. In rice, drought-tolerant quantitative trait loci in the chromosome segment substitution line (CSSL) ‘KDML105’ were co-localized with the markers of salt tolerance [4]. Comparison between the genomes of ‘CSSL104’ and ‘KDML105’ and co-expression network analysis of the 10 genes *CPFTSY*, *NDH-O*, *SOQ1*, *LHCB3*, *RRF*, *PGRL1B*, *HCF244*, *NAD(P)-linked oxidoreductase*, *LOC_Os11g43600*, and *MRL1*, have been identified to function in the acclimation of photosynthesis during drought stress. The analysis of seven T-DNA-tagged Arabidopsis mutant lines corresponding to seven of the ten genes (namely *ndhO* (*at1g74880*), *lhcb3* (*at5g54270*), *rrf* (*at3g63190*), *pgrl1b* (*at4g11960*), *pgrl1a* (*at4g22890*), *at2g27680*, and *mrl1* (*at4g34830*), revealed that all the genes, except *pgrl1a*, contributed to drought tolerance [5]. Salt tolerance has been investigated in rice CSSLs. It was reported that CSSL16 exhibited salt tolerance at the seedling and tillering stages, with several photosynthesis-related genes predicted to play roles in salt tolerance, including *OsPsbS1*, which encodes the chlorophyll *a*- and *b*-binding protein in the photosystem II, and *OsNDH-O*, which is involved in the adaptation of photosynthesis during drought stress in CSSL104 [5,6,7].

Chlorophyll fluorescence induction curve has been used to monitor the electron flow in plant photosynthetic systems, and it can be used to determine the difference in stress tolerance responses [8]. Therefore, we examined the chlorophyll fluorescence induction curves of normal and 3-(3,4-dichlorophenyl)-1,1-dimethylurea (DCMU) treated plants in normal and salt stress conditions to evaluate the energy flow in the photosystems.

In this study, we aimed to determine the roles in salt tolerance of the photosynthesis-related genes predicted to be involved in drought tolerance, namely, *NDH-O*, *LHCB3*, *RRF*, *PGRL1A*, *PGRL1B, LOC_Os11g43600*, and *MRL1*, using the T-DNA-tagged Arabidopsis mutant lines corresponding to these genes. Among the predicted genes involved in salt tolerance from CSSL16, *LOC_Os01g68450* has not been characterized [6]. Therefore, we incorporated Arabidopsis mutant line *at1g65230*, corresponding to *LOC_Os01g68450*, in this comparison. The most susceptible line among these mutants was further investigated and characterized by the complementation with the orthologous gene from rice to validate its role in salt tolerance.

## 2. Results

### 2.1. Mutation in At1g65230 Strongly Inhibited Plant Growth under Salt Stress

Arabidopsis mutant lines with T-DNA insertions at the orthologous rice genes were used to determine the total green area of the leaves when the plants were cultured under control and salt stress conditions.

**Figure 1:**
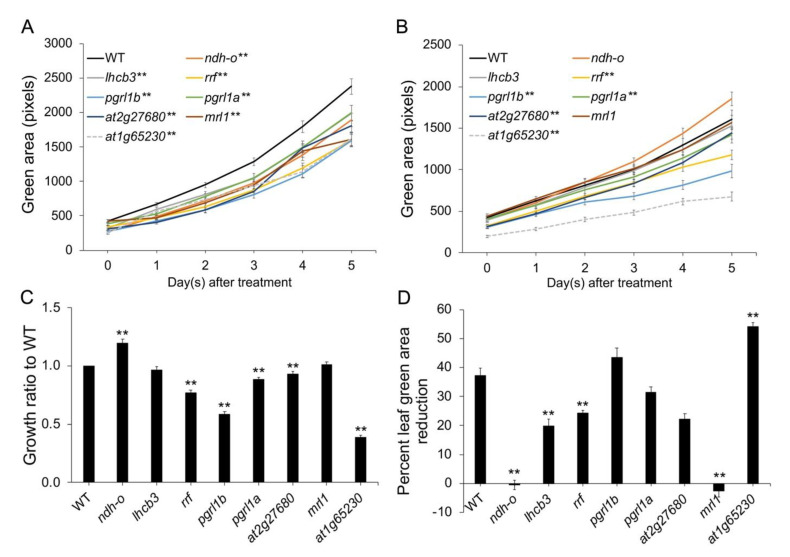
Total green area of the leaves in the Arabidopsis wildtype (WT) and mutant lines under the control conditions (**A**) and 75 mM NaCl stress (**B**). Relative growth rates of the Arabidopsis WT and mutant lines after five-day exposure to the control conditions (**C**) and 75 mM NaCl (**D**). ** represent highly significant differences in the means (*p* < 0.01), when compared to the WT using a *t*-test. Error bars show standard error and the experiment was performed with *n* = 48.

Under control conditions, the total green area in the leaves of all the Arabidopsis mutant lines were significantly lower than that of the wildtype (WT) (Figure 1A). Under salt stress, the green area in the leaves of the *ndho*, *lhbc3*, and *mrl1* mutants was similar to those of the WT under salt stress; however, the green area in the leaves of the remaining mutants was significantly lower than that of the WT (Figure 1B). Moreover, the green area in the leaves of the *at2g27680* mutant was significantly lower than that of the WT, but after five days of exposure to salt stress, it became similar to that of the latter. The *at1g65230* mutant exhibited the lowest green area in the leaves (Figure 1B), lowest growth ratio (Figure 1C), and maximum decrease in the leaf green area percentage (Figure 1D) compared with the WT under salt stress, suggesting the important role of *at1g65230* in adapting to salt stress. Therefore, the *at1g65230* mutant was selected for further characterization.

### 2.2. Ectopic Expression of LOC_Os01g68450 Reverted the Susceptibility of the At1g65230 Mutant to Salt Stress via the Light Reaction Adaptation

**Figure 2:**
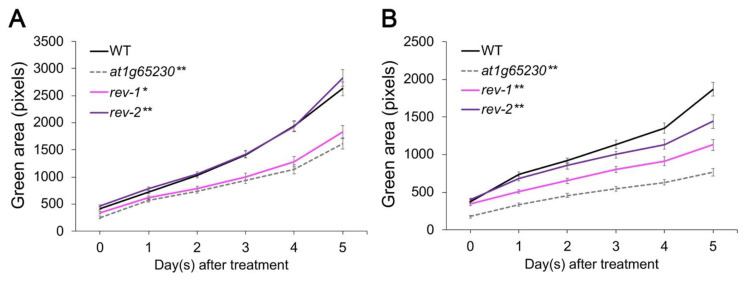
Total green area of the leaves in the Arabidopsis wildtype (WT) and revertant lines under the control conditions (**A**) and 75-mM NaCl stress (**B**). * represent the significant differences of the means (*p* < 0.05) compared to WT and ** represent the highly significant differences of the means (*p* < 0.01) compared to WT using a *t*-test. Error bars show standard error and the experiment was performed with *n* = 48.

To investigate the reversal of susceptibility of the *at1g65230* mutant to salt stress via the ectopic expression of *LOC_Os01g68450*, the ectopic expression construct was transformed into the *at1g65230* mutant. T_2_ homozygous expression lines, *rev-1* and *rev-2*, were used to determine the phenotype under the control and salt stress conditions. Both revertant lines showed very low expression of *at1g65230* but had detectable *LOC_Os01g68450* expression (Supplementary Figure S1). Both of the revertant lines grew better than the *at1g65230* mutant under control conditions. The revertant line rev-1 exhibited green area development similar to the WT under control conditions (Figure 2A), and both the revertant lines exhibited significantly higher green areas in the leaves than the mutant line under salt stress. However, after five days of exposure to salt stress, the revertant lines exhibited a significantly lower green area in the leaves than the WT (Figure 2B).

**Figure 3:**
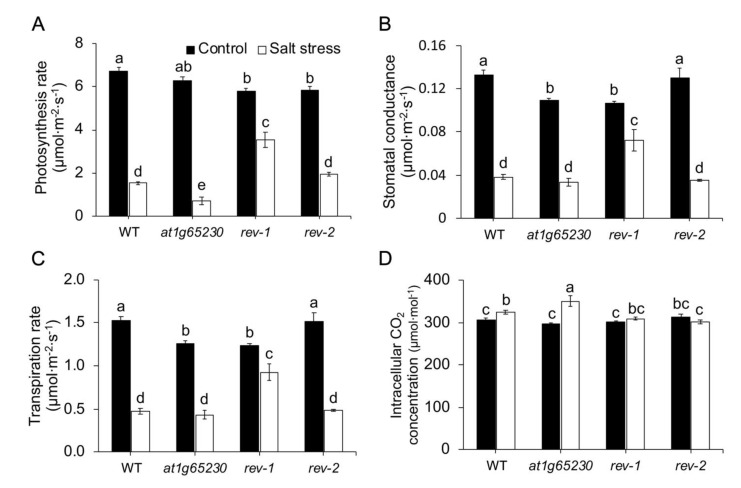
Photosynthetic parameters of the Arabidopsis wildtype (WT), *at1g65230* mutant, and revertant line (*rev-1 and rev-2*) cultured under control (black bars) and salt-stress (white bars) conditions. Photosynthetic rate (*P_n_*) (**A**), stomatal conductance (*g_s_*) (**B**), transpiration rate (*E*) (**C**), and intracellular CO_2_ concentration (*C_i_*) (**D**) were determined after 10 days of treatments. The different letters above the bars represent the significant difference among means (*p* < 0.05). Error bars show the standard error of 3 replicates.

Under normal growing conditions, the net photosynthetic rates (*P_n_*) of the mutant and revertant lines were similar but both the revertant lines had a significantly lower *P_n_*, than WT. Salt stress caused *P_n_* reduction in all lines. The mutant line had significantly lower *P_n_* than WT. However, both *rev-1* and *rev-2* had a significantly higher *P_n_* than the mutant line under salt stress (Figure 3A). Salt stress also caused a decrease in stomatal conductance (*g_s_*) in all lines. Similar levels of *g_s_* were detected in all lines, except *rev-1*, which showed a better ability to maintain *g_s_* under salt stress conditions (Figure 3B). The transpiration rate (*E*) pattern was similar to *g_s_* response (Figure 3C). These led to the highest concentration of intracellular CO_2_ (*C_i_*) being in the mutant line under salt stress conditions. Salt stress also caused a significant increase in *C_i_* in WT, but in both revertant lines, *C_i_* was maintained at the same level as detected under the normal condition (Figure 3D).

**Figure 4:**
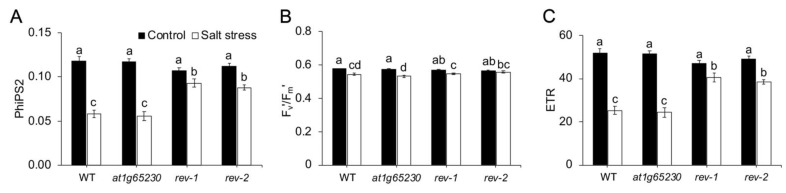
Efficiency of the light reaction in the Arabidopsis wildtype (WT), *at1g65230* mutant, and revertant line (*rev-1* and *rev-2*) determined after 10 days of treatment under control and salt stress conditions. Operating efficiency of PSII (**A**), excitation capture efficiency (**B**), and electron transport rate (**C**). The different letters above the bars represent the significant difference among means (*p* < 0.05). Error bars show the standard error of 3 replicates.

Based on *P_n_* values that were significantly higher in both revertant lines compared to the mutant line, the light reaction efficiency was investigated to determine the contribution of light reaction adaptation to salt stress in these lines. The operating efficiency of photosystem II (ΦPSII) of all lines grown in normal conditions were similar but decreased under salt stress condition. However, under salt stress, a ΦPSII reduction greater than 50% was detected in the WT and mutant lines, while a less than 20% reduction of ΦPSII was found in both revertant lines (Figure 4A). Under normal growth conditions, all lines exhibited similar excitation capture efficiency (F_v_’/F_m_’). The F_v_’/F_m_’ was detected as having been significantly decreased in salt-stressed WT, mutant, and *rev-1*; in salt-stressed *rev-2,* it was similar to the one grown in normal conditions (Figure 4B). Salt stress affected electron transport rate (ETR) in all the lines in a similar pattern as shown in ΦPSII responses (Figure 4C).

**Figure 5:**
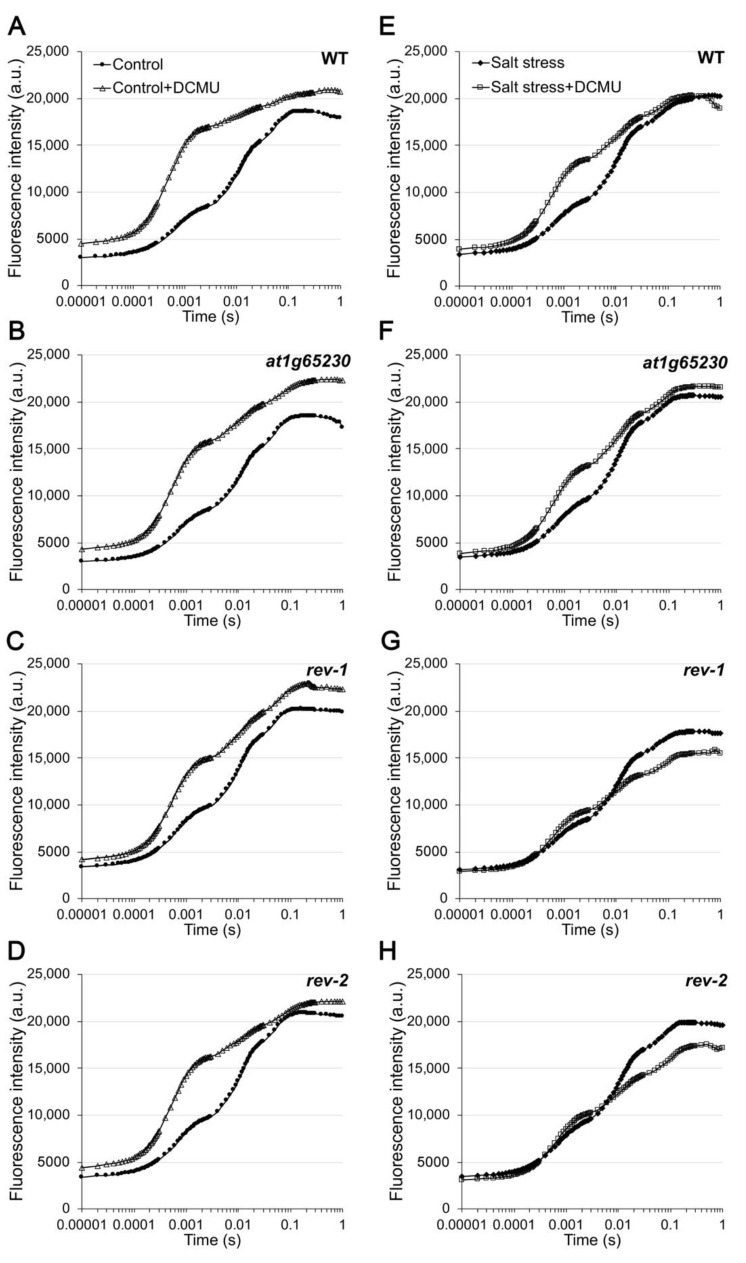
Chlorophyll fluorescence induction curve at 1 μs to 1 s in the Arabidopsis wildtype (WT), *at1g65230* mutant, and revertant line (*rev-1* and *rev-2*) determined after 10 days of treatment under control (**A**–**D**) and salt-stress (**E**–**H**) conditions. The trend lines show means of 6 replicates.

Chlorophyll fluorescence induction curves of the normal grown and salt-stressed plants were investigated to determine the effect of the mutated *at1g65230* gene and the ectopic expression of *LOC_Os01g68450* gene in the revertant lines. In normal conditions, the minimal fluorescence (F_0_) of all lines was similar. The mutation at the *at1g65230* gene showed no effects on the O–J–I–P curve when compared to that of WT (Figure 5A,B). The ectopic expression of the *LOC_Os01g68450* gene in an *at1g65230* background resulted in a slight increase in F_J_ and F_M_ (Figure 5C,D). When the light reaction was inhibited by the DCMU, the OJ curve was detected in all lines, each with a high F_J_, compared to the curve detected in non-DCMU-treated ones. DCMU treatment increased the F_0_ of all lines under normal conditions (Figure 5A–D).

Salt stress had no effect on *F*_0_ and *F*_J_ of all lines, but it increased *F*_M_ in WT and the mutant lines (Figure 5E,F); furthermore, lower *F*_M_ was found in *rev-1* (Figure 5G) and no effect on *F*_M_ was found in *rev-2* (Figure 5H). When salt-stressed plants were treated with DCMU, *F*_0_ was consistent with *F*_0_ of non-DCMU-treated ones in all lines. The *F*_J_ of WT and the mutant were increased (Figure 5E,F), but were lower than the *F*_J_ of the DCMU treated (*F*_J, DCMU_) plants grown in normal conditions (Figure 5A,B). In contrast, DCMU did not affect the *F*_J_ of the revertant lines under salt stress conditions (Figure 5G,H). DCMU had no significant effect on *F*_M_ of WT and the mutant line under salt stress, while the lower *F*_M_ was found in the DCMU treated (*F*_M, DCMU_) revertant lines under salt stress conditions (Figure 5G,H).

### 2.3. Investigation of At1g65230 and LOC_Os01g68450 Genes on PsbD Protein Stability under Salt Stress Condition

**Figure 6:**
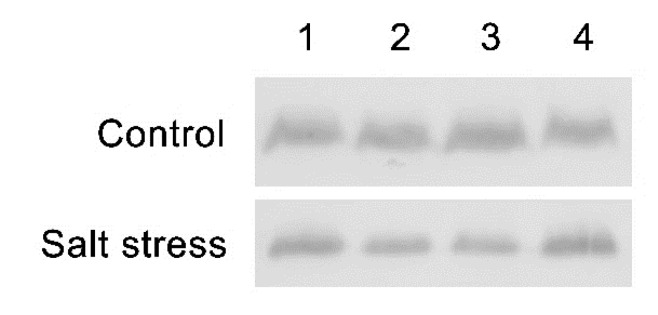
PsbD protein expression of the Arabidopsis wildtype (WT) (lane 1), *at1g65230* mutant (lane 2), and revertant line; *rev-1* (lane 3) and *rev-2* (lane 4) determined after 10 days of culturing under control and salt-stress conditions.

To investigate if the salt tolerance in the revertant lines was due to the ability to maintain PSII under salt stress conditions, PsbD protein, the main component of PSII proteins, was measured using a PsbD protein antibody. It was found that the mutation of the *at1g65230* gene and ectopic expression of the *LOC_Os01g68450* gene had no significant effect on PsbD protein content in both normal and salt stress conditions. However, the level of PsbD protein was decreased by salt stress treatment in all lines (Figure 6).

### 2.4. Ectopic Expression of LOC_Os01g68450 Gene Affected Photosynthetic Pigment Levels

According to the salt-tolerant phenotypes of the revertant lines, demonstrated by the maintenance of leaf green area and the photosynthetic ability under salt stress conditions, we selected *rev-2* as a representative to investigate the contents of photosynthetic pigment compared with WT and *at1g65230* mutant.

**Figure 7:**
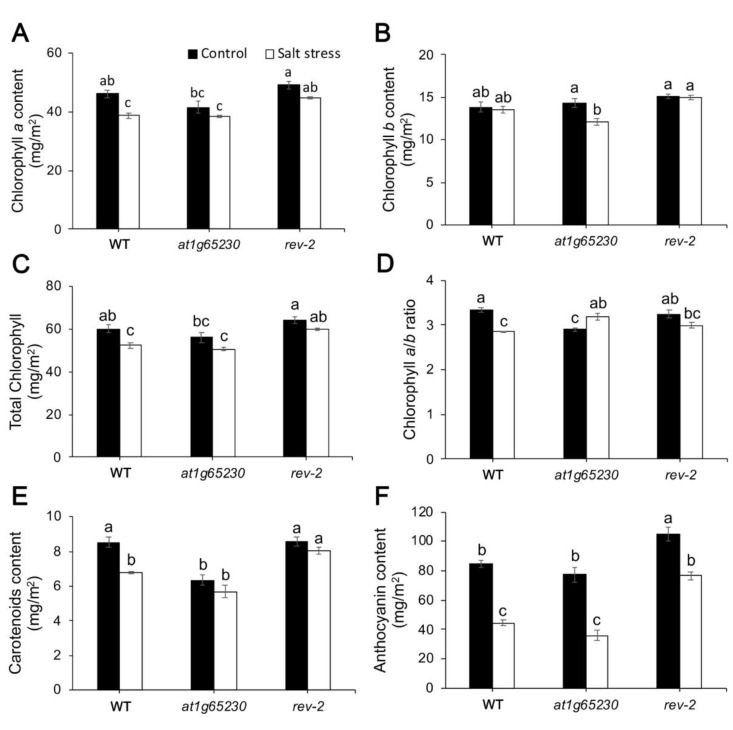
Pigment contents in the Arabidopsis wildtype (WT), *at1g65230* mutant, and revertant line (*rev-2*) determined after five days of culturing under control and salt-stress (200 mM NaCl) conditions: Chlorophyll *a* (**A**), chlorophyll *b* (**B**), total chlorophyll (**C**), chlorophyll *a*/*b* ratio (**D**), carotenoids (**E**), and anthocyanin (**F**). The different letters above the bars represent the significant difference among means (*p* < 0.05). Error bars show SE of 4 replicates and each replicate was performed with 4 samples.

Under control conditions, the mutation in the *at1g65230* gene tended to decrease total chlorophyll content especially chlorophyll *a* content, while the ectopic expression of *LOC_Os01g68450* gene showed no significant effects on chlorophyll contents (Figure 7A–C). Salt stress caused a significant reduction of chlorophyll *a* content in WT and *at1g65230* mutant, but a slight decrease in chlorophyll *a* content was detected in *rev-2* (Figure 7A). Chlorophyll *b* content in *rev-2* could be maintained under salt stress, leading to the significantly higher chlorophyll *b* and total chlorophyll content in *rev-2*, when compared to the mutant line (Figure 7B,C). The mutation in the *at1g65230* gene led to a significantly lower chlorophyll *a/b* ratio when compared to WT in normal grown conditions. The ectopic expression of the *LOC_Os01g68450* gene could revert the chlorophyll *a/b* ratio to a level similar to that in WT in normal grown conditions. In salt stress conditions, the chlorophyll *a/b* ratio was decreased, while it was increased in the mutant line. The ectopic expression of the *LOC_Os01g68450* gene could revert this response in the mutant line (Figure 7D).

The *at1g65230* mutant had significantly lower carotenoid content than WT and *rev-2* in control conditions. Salt stress caused the carotenoid content to become lower. However, the carotenoid content of *rev-2* was slightly decreased by salt stress, leading to significantly higher carotenoid content in *rev-2*, when compared to WT and *at1g65230* mutant (Figure 7E). Furthermore, anthocyanin content was also investigated. Ectopic expression of the *LOC_Os01g68450* gene led to the higher accumulation of anthocyanin content in both control and salt stress conditions when compared to WT and the mutant line. Salt stress caused the decline of anthocyanin content by 47.5% and 53.7% in WT and the mutant line, respectively, while anthocyanin content was decreased by 26.9 % in *rev-2* (Figure 7F).

## 3. Discussion

The growth responses of the selected mutant lines under salt stress were different from those shown under drought stress. According to a previous study, *ndhO*, *lhcb3, rrf*, and *at2g27680* mutants would exhibit higher growth rates than the WT under drought stress [5], because impairment in the components of the light-harvesting complex prevented chloroplast damage. However, this response was not observed in the *lhcb3* mutant under salt stress (Figure 1), possibly due to increased ion imbalance and toxicity. The knockout mutants of *RRF*, *PGRL1B*, *PGRL1A*, *At2g27680*, and *at1g65230* exhibited growth inhibition under both control and salt stress conditions, particularly the *at1g65230* mutant, which was highly susceptible to salt stress (Figure 1). This suggests that *at1g65230* has an important role in relation to salt tolerance.

**Figure 8:**
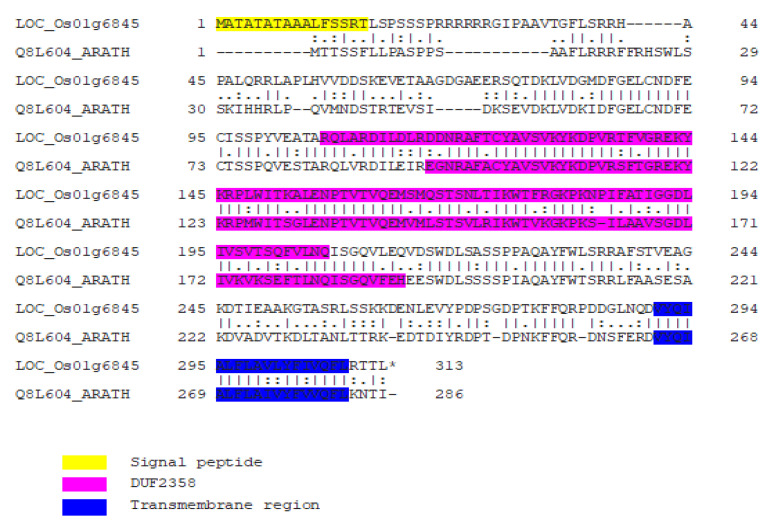
Amino acid sequence alignment of LOC_Os01g6845 and Q8L604, the protein product of *at1g65230*. Different motifs are indicated in yellow, pink, and blue. LOC_Os01g6845 sequence was obtained from the Rice Genome Annotation Project [9] and that of Q8L604 was obtained from The Arabidopsis Information Resource [10].

Rice *LOC_Os01g68450,* the homolog of *at1g65230*, encodes the protein Q8L604. Amino acid sequence alignment revealed 67.1% similarity between the two homologs. Furthermore, the DUF2358 and transmembrane domains were conserved between the two homologs, suggesting similar functions of their protein products (Figure 8). Thus, to validate the role of *LOC_Os01g68450*, it was ectopically expressed in the *at1g65230* mutant and its phenotype reversal ability was determined under salt stress. The *at1g65230* was already knocked out by T-DNA insertion and the ectopic expression of *LOC_Os01g68450* was revealed in both the revertant lines (Supplementary Figure S1).

Salt stress suppresses photosynthesis in various plant species, including rice (*Oryza sativa* L.) [11], chickpea (*Cicer arietinum* L.) [12], rocket (*Eruca sativa* L.) [13], and maize (*Zea mays* L.) [14]. Two independent revertant lines in Arabidopsis, with the ectopic expression of *LOC_Os01g68450*, had significantly higher green areas in the leaves and *P_n_* under salt stress, suggesting that *LOC_Os01g68450* increased salt tolerance in the *at1g65230* mutant ( Figure 2; Figure 3). Interestingly, *g_s_* of the mutant line and *rev-2* were similar (Figure 3B), although *rev-2* had a significantly higher *P_n_* (Figure 3A). This suggested that a low *P_n_* was not solely due to stomatal response to salt stress. The investigation of ΦPSII, F_v_’/F_m_’, and ETR suggested that the higher activity in light reaction in *rev-1* and *rev-2* demonstrated the better maintenance of ΦPSII. This could help the electron transport process in light reaction and contribute to the higher *P_n_* of the revertant lines under salt stress conditions. Plants have various mechanisms to regulate the process of gaseous exchange to adapt to salt-stress conditions [15]. The increase in carboxylation efficiency was detected as the salt tolerance phenotype in tomato and rice plants inoculated with salt-tolerant plant growth-promoting bacteria, which resulted in increased plant growth rates under salt stress. The increase in the ΦPSII, ETR, and carboxylation efficiency were observed in the salt-tolerant phenotypes [16]. Similar observations were reported in both *rev-1* and *rev-2* ( Figure 3; Figure 4), indicating that the ectopic expression of *LOC_Os01g68450* in the *at1g65230* mutant increased salt tolerance by enhancing the efficiency of the light-harvesting complex under salt stress. This study is the first to characterize the role of a protein with a DUF2358 domain on the enhancement of efficiency of the light-harvesting complex under salt stress.

The enhanced efficiency of PSII can be due to the ability to maintain the structure of a light-harvesting complex and/or the adaptation for energy transfer in PSII. Therefore, we investigated the stability of PsbD protein in PSII under salt stress conditions using the chlorophyll fluorescence induction curve (O-J-I-P test). Western blot analysis suggests that salt stress caused a decrease in the level of PsbD protein in all lines, but no significant difference in the PsbD protein level was detected therein (Figure 6), which in turn suggested that the ectopic expression of *LOC_Os01g68450* gene had no involvement on the expression or the stability of PSII under salt stress conditions.

The O-J-I-P test was used to investigate the changes in energy dissipation in PSII. Under normal conditions, the O-J-I-P of all lines were similar (Figure 5); under salt stress conditions, the *F*_M, DCMU_ of *rev-1,* and *rev-2* were lower than *F*_M_ (*F*_M_ of non-DCMU treated plants), while *F*_M, DCMU_ of WT and the mutant was similar to *F*_M_. It was suggested that this phenomenon was related to non-photochemical quenching (NPQ) [17]. Moreover, the increase in *F*_J, DCMU_ was detected only in WT and the mutant line, while both revertant lines showed that *F*_J_ was equal to *F*_J, DCMU_. DCMU typically prevents reoxidation of Q_A_^-^ by its displacement of Q_B_ at DI protein and leads to the increase in chlorophyll fluorescence at the J step [18]. This phenomenon could not be detected in both revertant lines, suggesting that the ectopic expression of *LOC_Os01g68450* provided another energy dissipation process, in PSII, to protect the overflow of absorbed light energy that can cause ROS and damage photosystem complexes. This could help maintain the efficiency of PSII and light reaction activity under salt stress conditions. The higher level of carotenoid content in the revertant lines, when compared with the WT and mutant lines, supported this hypothesis. The xanthophyll cycle plays an important role in NPQ [19]. The significantly higher carotenoid content (Figure 7) suggests that NPQ occurred in this response. Moreover, the anthocyanin content in the revertant line was almost 100% higher than the anthocyanin content in the salt-stressed-treated mutant. Anthocyanins function as antioxidants under stress conditions [20], indirectly preventing chlorophyll degradation and maintaining photosynthetic activity [21]. Carotenoids and anthocyanins also scavenge ROS that are generated in the cell after stress [22,23]. The overexpression of the gene encoding leucoanthocyanidin dioxygenase from *Reaumuria trigyna* Maxim. in Arabidopsis conferred abiotic stress tolerance through anthocyanin accumulation [24].

Considering these responses, the *LOC_Os01g68450* can be considered a salt-tolerant gene in rice as it plays a role in the light energy dissipation process during salt stress, thereby leading to the ability to maintain PSII efficiency and photosynthesis. This gene is located in the cluster with *PsbS1* gene on chromosome 1, which was previously reported as contributing to salt tolerance [6].

## 4. Materials and Methods

### 4.1. Plant Material

This study used the Arabidopsis ecotype Columbia-0 and mutant lines with T-DNA insertions in the genes of interest. The mutant lines SALK_097351, SALK_020314, SALK_015954, SALK_059238, SALK_133856, SALK_073120, SALK_060806, and SALK_130615 had the T-DNA inserted in the genes *at1g74880*, *at5g54270*, *at3g63190*, *at4g11960*, *at4g22890*, *at2g27680*, *at4g34830*, and *at1g65230*, respectively. All Arabidopsis mutant lines were obtained from the Arabidopsis Biological Resources Center [25].

### 4.2. Ectopic Expression and Revertant Line Generation

The cDNA of the rice ortholog of *at1g65230*, *LOC_Os01g68450* (Accession no: AK068538)*,* was cloned into the pJIM19 vector using the restriction enzymes *XbaI* and *XhoI* and T4 ligase. The plasmid was then introduced into *Agrobacterium tumefaciens* GV3101 using freeze–thaw transformation; kanamycin, rifampicin, and gentamicin were used as the selectable markers. The Col-0 ecotype and *at1g65230* mutant line were transformed using the floral dipping method to generate the revertant lines [6,7]. A completely randomized design (CRD) with 48 plants per line was used as the experimental design.

### 4.3. Estimating the Green Area of Leaves

The seeds of all the Arabidopsis lines were sterilized, placed on square plates, and kept undisturbed in a refrigerator for four days. The square plates were then placed in a growth chamber for germination at 22 °C/20 °C, 16/8 h light/dark cycle, 120 µmol photons of photosynthetically active radiation (PAR) m^−2^·s^−1^, and 60% humidity. After four days of germination, the seedlings were transferred to 48-well plates containing full Murashige and Skoog (MS) solid medium (control) or MS solid medium supplemented with 75 mM sodium chloride (salt stress) and grown under the same condition. The green areas of the leaves were estimated every day using a PlantScreen XYZ system (Photon Systems Instruments, Drásov, Czech Republic) [26].

### 4.4. Estimating Physiological Parameters

#### 4.4.1. Growth Conditions

The seeds of all Arabidopsis lines were soaked in distilled water and kept in dark for two days. The seeds were then sown in a soil mixture containing peat moss, perlite, and vermiculite (3:1:1). Plants were grown at 22 °C, 180 µmol photons of PAR m^−2^·s^−1^, and a 16/8 h light/dark cycle. Twenty-eight-day-old plants were treated with distilled water (control), 200 mM sodium chloride for five days, and 300 mM sodium chloride for five days (salt stress) and by withholding water (drought stress) The experiment was designed using a CRD with three replicates and four plants per replicate for each treatment.

#### 4.4.2. Photosynthetic Parameters

Photosynthetic parameters were estimated in the seventh leaf of all the Arabidopsis lines using an LI-6400XT portable photosynthesis system (LICOR, Lincoln, NE, USA) ten days after treatment [27]. *P_n_*, *C_i_*, *g_s_*, transpiration rate, Fv’/Fm’, ΦPSII, and ETR were estimated at 300 mmol^−1^ of flow of air m^−2^·s^−1^, 1000 mol  photons of PAR m^−2^ · s^−1^, 23–24 °C, and 400 mol·mol^−1^ of CO_2_.

#### 4.4.3. Photosynthetic Pigment and Anthocyanin Content

Leaf disks (diameter 7 mm) of all the Arabidopsis lines collected 5 days after treatment were soaked in 80% acetone and maintained at 14 °C for 72 h to extract chlorophylls and carotenoids. A spectrophotometer was used to measure the absorbance of the leaf extracts at A_470_, A_646.8_, and A_663.2_. The contents of the photosynthetic pigments were calculated using the following formulas [28]:

Chlorophyll *a* (Chl *a*) content = 12.25A_663.2_ − 2.79A_646.8_ (1)

Chlorophyll *b* (Chl *b*) content = 21.5A_646.8_ − 5.1A_663.2_ (2)

Total chlorophyll content = Chl *a* + Chl *b* (3)

Chlorophyll *a*/*b* ratio = Chl *a* / Chl *b* (4)

Total carotenoids = (100A_470_ − 1.82Chl *a* − 85.02chl *b*)/198 (5)

Anthocyanin extraction was performed using leaf disks soaked in 1% HCl in methanol and kept at 14 °C overnight in microcentrifuge tubes. After overnight soaking, 200 μL of milli-Q water and 500 µL of chloroform were added to each sample, which was then centrifuged at 16,000 x g for 3 min. The supernatant was discarded, 400 µL of 60% aqueous solution of 1% HCl in methanol was added to the pellet, and absorbance was measured at A_530_ and A_675_ using a microplate reader (Synergy^TM^ HTX Multi-Mode Microplate Reader, BioTex, Houston, TX, USA). The anthocyanin content in the leaves was calculated using the following formula [29]:

Anthocyanin content = (A_657_ − A_530_)/area (m^2^) (6)

#### 4.4.4. Chlorophyll Fluorescence Induction Curves

Chlorophyll fluorescence induction curves were estimated with a Pocket PEA (Hansatech instruments, Pentney, UK), using the fifth and seventh leaves of all Arabidopsis lines, ten days after treatment. The leaves were dark-adapted for 30 min before measurement at 3000 μmol·m^−2^·s^−1^. For DCMU treatment, the Arabidopsis plants were sprayed with 40 μM of DCMU and 0.1% Tween 20 1 h before measurement [30].

### 4.5. Quantification of Gene Expression

#### 4.5.1. RNA Extraction

The fresh fifth and sixth leaves of all Arabidopsis lines were collected after ten days of treatment. Tissues were frozen directly in liquid nitrogen. GENEzol^TM^ reagent (Geneaid, Taiwan) was used for RNA extraction. The total RNA concentration was measured using NanoDrop spectrophotometers (Thermo Fisher Scientific, Waltham, MA, USA). The RNA samples were treated with DNase I (Thermo Fisher scientific, USA) to remove DNA, following which the RNA was reverted to DNA using the iScript^TM^ cDNA synthesis kit (BIO-RAD, Hercules, CA, USA).

#### 4.5.2. Gene Quantification

A quantitative real-time polymerase chain reaction (RT-PCR) test was conducted using Luna qPCR master mix (NEB, Ipswich, MA, USA) with three technical replications for each sample. The primers used for qPCR were as follows:
at1g65230-FAATCCGCAAAAGATGTCGCCat1g65230-RGACAATGGCTAGAAACAGTGCAALOC_Os01g68450-FAGGGTACAGCATCTCGGCTALOC_Os01g68450-RCAGGAAGAGCGCAATCTGGTEF1α-FTGCCGCAGGTGAATCAAAGGEF1α-RCCCAATTACGAGAACAACGCTCTG

The PCR process was as follows: initial denaturation at 95 °C for 60 s followed by 40 cycles of denaturation at 95 °C for 15 s and extension at 61.5 °C for 30 s and 95 °C for 5 s. The melt curve is at 60–94 °C with a 5 °C increase every 5 s. The transcription levels were normalized using EF1α [31]. The relative expression levels were calculated using the ∆∆Ct method [32].

### 4.6. Immunoblotting

#### 4.6.1. Extraction of Thylakoid Membranes

The seventh and eighth leaves of all Arabidopsis lines were collected ten days after treatment and frozen directly in liquid nitrogen. The leaves were ground using Mixer Mill MM400 (RETSCH, Haan, Germany). Following this, 1 mL of ice-cold P1 buffer (50 mM HEPES pH 7.5, 5 mM MgCl_2_, 330 mM sorbitol, 10 mM NaF, 0.1% BSA, and 5 mM ascorbic acid) was added to the microcentrifuge tube with a sample in dim light and kept it on ice The samples were centrifuged at 1500 rpm, at 4 °C, for 1 min. The supernatants were transferred to new 1.5 mL microcentrifuge tube and centrifuged at 7000 rpm and 4 °C for 5 min. The supernatants were discarded and the pellets resuspended in 80 μL P2 buffer (50 mM HEPES pH 7.5, 5 mM MgCl_2_, and 10 mM NaF). Samples were again centrifuged at 7000 rpm, at 4 °C for 5 min, and the supernatants were discarded carefully. The pellets were then resuspended in 30 μL of P3 buffer (50 mM HEPES pH 7.5, 10 mM MgCl_2_, 100 mM sorbitol and 10 mM NaF) [33]. Finally, the samples were then analyzed in terms of chlorophyll concentration [28].

#### 4.6.2. Western Blot Analysis

Samples of the thylakoid membrane (60 μg/mL) were prepared for loading in 6× SDS sample buffer (reducing). The samples were incubated at 100 °C for 5 min, following which 10 μL of prepared sample was loaded into 4% Stacking gel and 12% resolving gel. The electrophoresis separation was performed at 100 V for 100 min using electrophoresis chamber (BIO-RAD, USA).

For blotting, PVDF membranes (BIO-RAD, USA) were pre-wetted in 100% methanol for 5 min. The gel, PVDF membranes, and 6 pieces of filter paper were then incubated in 1× transfer buffer for 30 m. The blotting sandwich was prepared using three pieces of filter paper, PVDF membrane, gel, and an additional three pieces of filter paper. Blotting was performed using Semi-Dry Blotter (Clever Scientific, Rugby, UK).

The membranes were blocked for 1 h with blocking buffer (1% low-fat milk powder in 1× TBS-T) after which the blocking buffer was discarded. The membranes were then incubated with 1:50,000 of PsbD primary antibody (Agrisera, Vännäs, Sweden) in 10 mL of blocking buffer for 1 h. Following this, the primary antibody was discarded and the membrane was washed with TBS-T, 3 times, for 5 min. The membrane was then incubated in 1:10,000 of HRP secondary antibody in 10 mL of blocking buffer for 1 h. The secondary antibody was again discarded and the membrane washed; the first 3 times with TBS-T, for 5 min, and then with TBS for 5 min. For signal detection, the membrane was incubated in DAB HRP substrate for 1 min; after signal detection, it was washed with distilled water [34].

### 4.7. Statistical Analyses

The images of the green area of the leaves were analyzed using MATLAB (R2015; MathWorks Inc., Natick, MA, USA). Independent *t*-tests were performed using SPSS version 22 (IBM, Armonk, NY, USA). Analysis of variance and Duncan’s multiple range test were used to analyze the photosynthetic parameters and pigment contents with three replicates.

## 5. Conclusions

Based on this study, *LOC_Os01g68450* confers salt tolerance to plants by providing an energy dissipation that is appropriate to maintain PSII efficiency, leading to the maintenance of growth and the net photosynthesis rate. The accumulation of carotenoids and anthocyanins can play roles in protecting the light-harvesting complex and increasing the photosynthetic ability of plants under salt stress. The *LOC_Os01g68450* gene is located in salt tolerance QTL previously reported by Kanjoo et al. [4]. Therefore, it suggested that this salt tolerance QTL supports the photosynthesis adaptation under salt stress conditions and can be a target region for rice breeding programs in the future.

## Data Availability

The data presented in this study are available in this article or supplementary material here.

## Supplementary Materials

The following supporting information can be downloaded at: https://www.mdpi.com/article/10.3390/plants11091233/s1, Figure S1: Relative expression of *At1g65230* and *LOC_Os01g68450* in the Arabidopsis wildtype (WT), at1g65230 mutant, and revertant line (*rev-1* and *rev-2*) determined after ten days of culturing under control and salt-stress conditions.
