# Supplementary material for: Role of LOC_Os01g68450, Containing DUF2358, in Salt Tolerance Is Mediated via Adaptation of Absorbed Light Energy Dissipation
Source: Plants (Basel). 2022 May 2;11(9):1233. doi: 10.3390/plants11091233 (PMC9105198; doi:10.3390/plants11091233)
Supplement: Supplementary file 1 [file plants-11-01233-s001.zip › plants-1671523-supplementary.pdf]

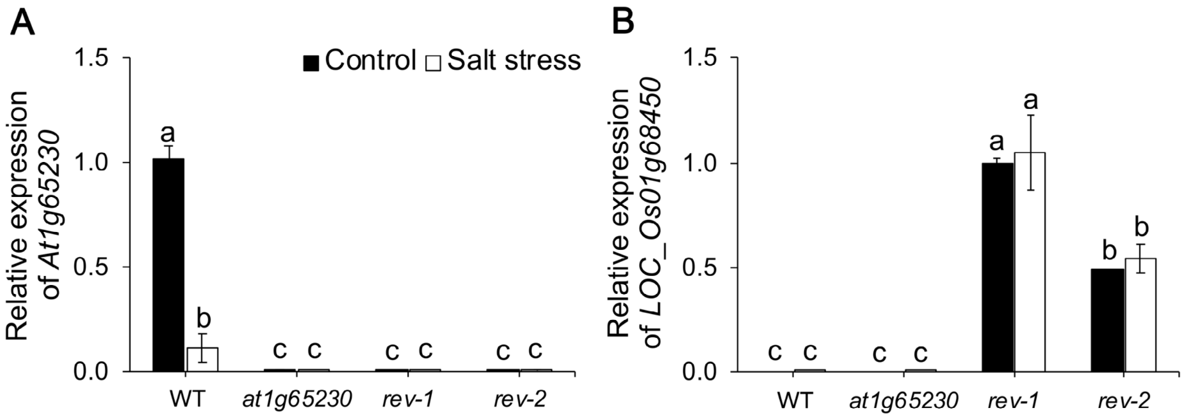

Supplementary Figure S1 Relative expression of *At1g65230* and *LOC_Os01g68450* in the Arabidopsis wildtype (WT), at1g65230 mutant, and revertant line (rev-1 and rev-2) determined after ten days of culturing under control and salt –stress conditions: expression of *AT1g65230* (**A**) and expression of *LOC_Os01g68450* (**B**). The different letters above the bars represent the difference among means (p < 0.05). Error bars show of 3 replicates.
